# Uso de herramientas tecnológicas: un reto para la educación de enfermería*

**DOI:** 10.15649/cuidarte.3310

**Published:** 2023-12-25

**Authors:** Javier Mauricio Sánchez-Rodríguez, Luisa Fernanda Moscoso-Loaiza

**Affiliations:** 1 . Facultad de Enfermería, Fundación Universitaria Sanitas, Bogotá, Colombia. E-mail: jmsanchezro@unisanitas.edu.co Facultad de Enfermería Fundación Universitaria Sanitas Bogotá Colombia jmsanchezro@unisanitas.edu.co; 2 . Facultad de Enfermería, Fundación Universitaria Sanitas, Bogotá, Colombia. E-mail: lfmoscosolo@unisanitas.edu.co Facultad de Enfermería Fundación Universitaria Sanitas Bogotá Colombia lfmoscosolo@unisanitas.edu.co

Estimada editora,

Desde el 2004 la Organización de las Naciones Unidas para la Educación, la Ciencia y la Cultura (UNESCO) ha resaltado la importancia de la incorporación de las tecnologías de la información y la comunicación (TIC) en los diferentes procesos de enseñanza- aprendizaje, como consecuencia de su rápido y creciente desarrollo, ya que se consideran relevantes como complementos pedagógicos en los diferentes niveles de formación, especialmente en los de educación superior. Para su implementación, es indispensable disponer de las herramientas suficientes para el fortalecimiento de las competencias digitales, el establecimiento de resultados de aprendizaje, así como el interés y motivación de los diferentes actores, principalmente estudiantes y docentes^1^.

En este sentido, el uso de TIC en el proceso de formación de profesionales de enfermería ha promovido la innovación en el aula en el proceso de enseñanza-aprendizaje, fortaleciendo el desarrollo de habilidades principalmente enfocadas en la práctica profesional, el pensamiento crítico, y la articulación teórico-practica, apoyando la flexibilidad curricular^2^.

En la revisión integrativa de Girao et. al., se agrupan las TIC implementadas para la educación en enfermería en tres ejes centrales; el primero hace referencia a las tecnologías desarrolladas para el uso en aulas presenciales, ubicando herramientas multimedia para trabajos colaborativos, estudio, presentaciones, y evaluaciones principalmente; incluyendo también el uso de diferentes dispositivos para realizar inmersiones profundas, por ejemplo, con realidad aumentada. El segundo grupo se enfoca en plataformas online para la enseñanza a distancia, las cuales están orientadas hacia la complementación de las actividades desarrolladas en las aulas presenciales promoviendo ambientes digitales de aprendizaje y actividades e-learning. El tercer eje relaciona las TIC en los abordajes para la práctica de simulaciones realistas, este grupo se enfoca en la incorporación de recursos tecnológicos para las clases teórico-prácticas, a través del uso de casos clínicos, fomentando la toma de decisiones, el juicio clínico, el trabajo interdisciplinar, siendo clave el fomento de la relación enfermero-paciente y la práctica del debriefing y la autorreflexión como parte de la evaluación de los aprendizajes^3^.

Estos hallazgos permiten evidenciar como las TIC son aliadas en el proceso de adquisición de competencias no solo centradas en el hacer, sino también en el saber, en función principalmente de su aprovechamiento, ya que permite el desarrollo de capacidades creativas, analíticas y de innovación, características que son necesarias para afrontar las demandas del mundo actual.

De otra parte, Correa y Martínez han identificado barreras que limitan la implementación de las TIC en educación superior, clasificándolas en tres diferentes niveles. Un nivel micro, en el que se ubican aquellas relacionadas con los docentes, asociadas con sus habilidades y competencias digitales por lo que, se requiere de formación continua dirigida a la actualización y adquisición de habilidades digitales, con el fin de que se logren incorporar las herramientas tecnológicas en los procesos de enseñanza con un propósito claro, reconociendo sus bondades, y no solo como un requisito que demandan los sistemas educativos.

A nivel meso, se ubican las barreras institucionales, siendo necesario tanto disponer de recursos e infraestructura suficientes, como facilitar el acceso a las tecnologías por parte de los actores principales del proceso. Y en el tercer y último nivel, denominado macro, se enmarca a los sistemas educativos en los que se desarrollan los procesos de enseñanza aprendizaje, los cuales deben reconocer la necesidad de reformar e innovar en las formas de evaluación, considerando las competencias obtenidas frente a las requeridas en la incorporación de las TIC en la práctica docente^4^.

A raíz del confinamiento por COVID-19, la educación en enfermería al igual que otras disciplinas y en especial las relacionadas con el área de las ciencias de la salud, vivieron un momento coyuntural para la formación de futuros profesionales, por lo que se tuvo que acelerar o incluso verse en la obligación, de la implementación de las TIC para asegurar los procesos de enseñanza-aprendizaje, en este sentido, aquellas instituciones en donde se tenían avances importantes en esta área, reportaron una mejor adaptación ante la necesidad de escenarios en su totalidad virtuales; por lo cual, es importante tomar en cuenta las lecciones aprendidas que dejó la educación en tiempos de pandemia, como lo es la necesidad de fortalecer las habilidades digitales, que es posible la innovación en el aula, algunas temáticas pueden desarrollarse mediadas por tecnología, los escenarios simulados siguen siendo un apoyo en el logro de competencias prácticas, el fomento de la autorregulación es clave para el aprendizaje al incorporar las TIC, la preocupación de los diferentes actores educativos no debe girar únicamente en el logro de competencias y formación académica, sino que también hay que tener en cuenta la salud mental de docentes y estudiantes, entre otras^5^^-^^7^, en especial al momento de considerar la implementación de escenarios híbridos de aprendizaje o realizar ajustes curriculares para incluir contenidos mediados por tecnología en los programas de enfermería.

Otro de los temas emergentes que está representando un reto importante para la formación de futuros profesionales, es el avance en relación con las inteligencias artificiales (IA), donde convergen la integración de las TIC, la informática en la educación, los procesos de investigación y la práctica clínica; donde si bien se puede realizar una mala praxis, como por ejemplo, el uso de IA tipo ChatGPT para la entrega y presentación de trabajos por parte de los estudiantes va a limitar el desarrollo del pensamiento crítico y creativo, y por parte de los docentes presentará limitaciones al momento de evaluar su proceso de aprendizaje debido a esta práctica inadecuada. No obstante, el uso de las IA no se puede estigmatizar en la incorporación de escenarios de enseñanza-aprendizaje, en especial desde la formación en enfermería, dado que se proyectan diversas innovaciones en este campo, como lo comenta Mejías y colaboradores en su revisión, en aspectos como la mejorar los escenarios virtuales introduciendo avatares, chatbots de tutores, así como también, para la gestión en la práctica y la atención al paciente, es decir que las IA en salud están cambiando el paradigma de la educación, lo que requerirá preparación, adopción y disposición por parte de los educadores^8^.

Finalmente, hay resaltar que, si bien los diferentes avances en las TIC complementan la formación de futuros profesionales de enfermería, para el logro de competencias y resultados de aprendizaje; por si solas estas no garantizan el proceso de enseñanza-aprendizaje; de otra parte, teniendo en cuenta el estado actual, las practicas presenciales no pueden ser reemplazadas aun por las TIC, debido a que una de las características principales en la formación es el desarrollo de habilidades comunicativas e interacción con los sujetos de cuidado, por tanto, es necesario por parte de los docentes e instituciones seguir fortaleciendo el pensamiento crítico, el desarrollo de habilidades para la toma de decisiones y la calidad académica.

## References

[1] Khvilon E, Patru M (2004). Las Tecnologías de La Información y La Comunicación En La Formación Docente: Guía de Planificación.

[2] Salvador PTC de O, Martins CCF, Alves KYA, Pereira MS, Santos VEP, Tourinho FSV (2015). Tecnología no ensino de enfermagem. Rev Baiana Enfermagem.

[3] Araújo-Girao AL, Cavalcante MLSN, Oliveira ICL, Freitas-Aires S, Oliveira SKP, Carvalho REFL (2020). Tecnologías en la enseñanza en enfermería, innovación y uso de TICs: revisión integrativa. Enfermería Universitaria.

[4] Correa Gorospe JM, Martínez Arbelaiz A (2010). ¿Qué hacen las escuelas innovadoras con la tecnología?: las TIC al servicio de la escuela y la comunidad en el Colegio Amara Berri. Teoría.. Educ Educ Cult En Soc Inf..

[5] Deng J, Zhou F, Hou W, Silver Z, ChY Wong, Chang O (2021). The prevalence of depressive symptoms, anxiety symptoms and sleep disturbance in higher education students during the COVID-19 pandemic: A systematic review and meta-analysis. Psychiatry Res..

[6] Vilar Pont M, Salgado Rodríguez MC, Paradell Blanc N, Pinsach Bosh L (2021). Impacto de la implementación de las nuevas tecnologías para innovar y transformar la atención primaria:la enfermera tecnológica. Aten Primaria Práctica..

[7] Magaña L, Biberman D (2022). Training the Next Generation of Public Health Professionals. Am J Public Health..

[8] Mejías M, Guarate Coronado YC, Jiménez Peralta AL (2022). Inteligencia artificial en el campo de la enfermería. Implicaciones en la asistencia, administración y educación. Salud Cienc Tecnol..

